# Lossless Transformations and Excess Risk Bounds in Statistical Inference

**DOI:** 10.3390/e25101394

**Published:** 2023-09-28

**Authors:** László Györfi, Tamás Linder, Harro Walk

**Affiliations:** 1 Department of Computer Science and Information Theory, Budapest University of Technology and Economics, H-1111 Budapest, Hungary; gyorfi@cs.bme.hu; 2 Department of Mathematics and Statistics, Queen’s University, Kingston, ON K7L 3N6, Canada; 3 Fachbereich Mathematik, Universität Stuttgart, 70569 Stuttgart, Germany; harro.walk@mathematik.uni-stuttgart.de

**Keywords:** statistical inference with loss, strongly consistent test, information-theoretic bounds, classification, regression, portfolio selection, information bottleneck, deep learning

## Abstract

We study the excess minimum risk in statistical inference, defined as the difference between the minimum expected loss when estimating a random variable from an observed feature vector and the minimum expected loss when estimating the same random variable from a transformation (statistic) of the feature vector. After characterizing lossless transformations, i.e., transformations for which the excess risk is zero for all loss functions, we construct a partitioning test statistic for the hypothesis that a given transformation is lossless, and we show that for i.i.d. data the test is strongly consistent. More generally, we develop information-theoretic upper bounds on the excess risk that uniformly hold over fairly general classes of loss functions. Based on these bounds, we introduce the notion of a δ-lossless transformation and give sufficient conditions for a given transformation to be universally δ-lossless. Applications to classification, nonparametric regression, portfolio strategies, information bottlenecks, and deep learning are also surveyed.

## 1. Introduction

We consider the standard setting of statistical inference, where *Y* is a real random variable, having a range Y⊂R, which is to be estimated (predicted) from a random observation (feature) vector *X*, taking values in $R^{d}$. Given a measurable predictor $f:R^{d}\to Y$ and measurable loss function $\ell :Y\times Y\to R_{+}$, the loss incurred is ℓ(Y,f(X)). The minimum expected risk in predicting *Y* from the random vector *X* is

$$L_{\ell }^{*}\left(Y|X\right)=\underset{f:R^{d}\to Y}{\inf}E\left[\ell \left(Y,f\left(X\right)\right)\right],$$

where the infimum is over all measurable *f*.

Suppose that the tasks of collecting data and making the prediction are separated in time or in space. For example, the separation in time happens when the data are collected first and the statistical modeling and analysis are performed much later. Separation in space can be due, for example, to collecting data at a remote location and making predictions centrally. Such situations are modeled using a transformation $T:R^{d}\to R^{d^{\prime }}$, so that the prediction regarding *Y* is made from the transformed observation T(X), instead of *X*. An important example for such a transformation is quantization, in which case T(X) is a discrete random variable. Clearly, one always has $L_{\ell }^{*}\left(Y|T\left(X\right)\right)\geq L_{\ell }^{*}\left(Y|X\right)$. The difference $L_{\ell }^{*}\left(Y|T\left(X\right)\right)-L_{\ell }^{*}\left(Y|X\right)$ is sometimes referred to in the literature as excess risk. A part of this paper is concerned with transformations for which the excess risk is zero, no matter the underlying loss function *ℓ*. Such transformations are universally lossless, in the sense that they can be chosen before the cost function *ℓ* for the underlying problem is known. More formally, we can state the following definition.

**Definition** **1** (lossless transformation)**.** *For a fixed joint distribution of Y and X, a (measurable) transformation $T:R^{d}\to R^{d^{\prime }}$ is called* universally lossless *if for any loss function $\ell :Y\times Y\to R_{+}$ we have*

$$L_{\ell }^{*}\left(Y|T\left(X\right)\right)=L_{\ell }^{*}\left(Y|X\right).$$

An important special transformation is feature selection. Formally, for the observation (feature) vector $X=(X^{\left(1\right)},\ldots ,X^{\left(d\right)})$ and S⊂{1,…,d}, consider the |S|-dimensional vector $X_{S}=\left(X^{\left(i\right)},i\in S\right)$. Typically, the dimension |S| of $X_{S}$ is significantly smaller than *d*, the dimension of *X*. If we have

$$L_{\ell }^{*}\left(Y|X_{S}\right)=L_{\ell }^{*}\left(Y|X\right),$$

for all loss functions *ℓ*, then the feature selector $X\mapsto X_{S}$ is universally lossless. For fixed loss *ℓ*, the performance of any statistical inference method is sensitive to the dimension of the feature vector. Therefore, dimension reduction is crucial before choosing or constructing an inference method. If $X_{S}$ is universally lossless, then the complement feature subvector $X_{S^{c}}$ is irrelevant. It is an open research problem how to efficiently search a universally lossless $X_{S}$ with minimum size |S|. Since, typically, the distribution of the pair *X* and *Y* is not known and must be inferred from data, any such search algorithm needs a procedure for testing for the universal losslessness property of a feature selector.

In the first part of this paper, we give a necessary and sufficient condition for a given transformation *T* to be universally lossless and then construct a partitioning-based statistic for testing this condition if independent and identically distributed training data are available. With the null hypothesis being that a given transformation is universally lossless, the test is shown to be strongly consistent, in the sense that it almost surely (a.s.) makes finitely many Type I and II errors.

In many situations, requiring that a transformation *T* is universally lossless is too demanding. The next definition relaxes this requirement.

**Definition** **2** (δ-lossless transformation)**.** *For a fixed joint distribution of Y and X, and δ>0, a transformation $T:R^{d}\to R^{d^{\prime }}$ is called* universally δ-lossless *with respect to a class of loss functions L, if we have*

$$L_{\ell }^{*}\left(Y|T\left(X\right)\right)-L_{\ell }^{*}\left(Y|X\right)\leq \delta \;for\;all\;\ell \in L.$$

In the second part of this paper, we derive bounds on the excess minimum risk $L_{\ell }^{*}\left(Y|T\left(X\right)\right)-L_{\ell }^{*}\left(Y|X\right)$ in terms of the mutual information difference I(Y;X)−I(Y;T(X)) under various assumptions about *ℓ*. With the aid of these bounds, we give information-theoretic sufficient conditions for a transformation *T* to be δ-lossless with respect to fairly general classes of loss functions *ℓ*. Applications to classification, nonparametric regression, portfolio strategies, the information bottleneck method, and deep learning are also reviewed.

*Relationship with prior work*

Our first result, Theorem 1, which shows that a transformation is universally lossless if and only if it is a sufficient statistic, is likely known, but we could not find it in this explicit form in the literature (a closely related result is the classical Rao–Blackwell theorem of mathematical statistics, e.g., Schervish ([1], Theorem 3.22)). Due to this result, testing with independent data whether or not a given transformation is universally lossless turns into a test for conditional independence. Our test in Theorem 2 is based on the main results in Györfi and Walk [2], but our construction is more general and we also correct an error in the proof of ([2], Theorem 1). Apart from [2], most of the results in the literature of testing for conditional independence are for real-valued random variables and/or assume certain special distribution types, typically the existence of a joint probability density function. Such assumptions exclude problems where *Y* is discrete and *X* is continuous, as is typical in classification, or problems where the observation *X* is concentrated on a lower dimensional subspace or manifold. In contrast, our test construction is completely distribution free and its convergence properties are also (almost) distribution free. A more detailed review of related work is given in Section 2.1.

The main result in Section 3 is Theorem 3, which bounds the excess risk in terms of the square root of the mutual information difference I(Y;X)−I(Y;T(X)). There is a history of such bounds, possibly starting with Xu and Raginsky [3], where the generalization error of a learning algorithm was upper bounded using constant times the square root of the mutual information between the hypothesis and the training data (see also the references in [3,4]). This result has since been extended in various forms, mostly concentrating on providing information-theoretic bounds for the generalization capabilities of learning algorithms, instead of looking at the excess risk; see, e.g., Raginsky et al. [5], Lugosi and Neu [6], Jose and Simeone [7], and the references therein, just to mention a few of these works. The most relevant recent work relating to our bounds in Section 3 seems to be Xu and Raginsky [4], where, among other things, information-theoretic bounds were developed on the excess risk in a Bayesian learning framework; see also Hafez-Kolahi et al. [8]. The bounds in [4] are not on the excess risk $L_{\ell }^{*}\left(Y|T\left(X\right)\right)-L_{\ell }^{*}\left(Y|X\right)$; they involve training data, but their forms are similar to ours. It appears that our Theorem 3 gives a bound that holds uniformly for a larger class of loss functions *ℓ* and joint distributions of *Y* and *X*; however, in [4], several other bounds are presented that are tighter and/or allow more general distributions, for specific fixed loss functions.

*Organization*

This paper is organized as follows. In Section 2, we characterize universally lossless transformations and introduce a novel strongly consistent test for the property of universal losslessness. In Section 3, information-theoretic bounds on the excess minimum risk are developed and are used to characterize the δ-losslessness property of transformations. Section 4 surveys connections with, and applications to, specific prediction problems, as well as the information bottleneck method in deep learning. The somewhat lengthy proof of the strong consistency of the test in Theorem 2 is given in Section 5. Concluding remarks are given in Section 6.

## 2. Testing the Universal Losslessness Property

In this section, we first give a characterization of universally lossless transformations for a given distribution of the pair (X,Y). In practice, the distribution of (X,Y) may not be known, but a sequence of independent and identically distributed (i.i.d.) copies of (X,Y) may be available. For this case, we construct a procedure to test if a given transformation is universally lossless and prove that, under mild conditions, the test is strongly consistent.

### 2.1. Universally Lossless Transformations

Based on Definition 1, we introduce the null hypothesis

(1) $$\begin{array}{c} H_{0}=\left\{T:\mathrm{the}\;\mathrm{transformation}T\mathrm{is}\;\mathrm{universally}\;\mathrm{lossless}.\right\} \end{array}$$

A transformation (statistic) T(X) is called sufficient if the random variables *Y*, T(X), *X* form a Markov chain in this order, denoted by Y→T(X)→X (see, e.g., Definition 3.8 and Theorem 3.9 in Polyanskiy and Wu [9]).

For a binary valued *Y*, Theorems 32.5 and 32.6 from Devroye et al. [10] imply that the statistic T(X) is universally lossless if, and only if, it is sufficient. The following theorem extends this property to general *Y*. This result is likely known, but we could not find it in the given form.

**Theorem** **1.** *The transformation T is universally lossless if, and only if, Y→T(X)→X is a Markov chain.*

**Proof.**  Assume first that Y→T(X)→X is a Markov chain. This is equivalent to having P(Y∈A|X,T(X))=P(Y∈A|T(X)) almost surely (a.s.) for any measurable A⊂Y. Then we have

$$\begin{array}{cc} L_{\ell }^{*}\left(Y|X,T\left(X\right)\right)\; & =E\left[\underset{y\in Y}{\inf}E\left[\ell \left(Y,y\right)|X,T\left(X\right)\right]\right]\\ \; & =E\left[\underset{y\in Y}{\inf}E\left[\ell \left(Y,y\right)|T\left(X\right)\right]\right]\\ \; & =L_{\ell }^{*}\left(Y|T\left(X\right)\right). \end{array}$$

Since $L_{\ell }^{*}\left(Y|X,T\left(X\right)\right)\leq L_{\ell }^{*}\left(Y|X\right)\leq L_{\ell }^{*}\left(Y|T\left(X\right)\right)$ always holds, we obtain $L_{\ell }^{*}\left(Y|T\left(X\right)\right)=L_{\ell }^{*}\left(Y|X\right)$ for all *ℓ*, so T(X) is universally lossless.

Now, assume that the Markov chain condition Y→T(X)→X does not hold. Then, there exists a measurable A⊂Y with 0<P(Y∈A)<1 and $B\subset R^{d}$ with P(X∈B)>0, such that

P(Y∈A|X,T(X))≠P(Y∈A|T(X))ifX∈B.

Let $h\left(y\right)=I_{y\in A}$, where $I_{E}$ is the indicator function of event *E*, and define the binary valued $\hat{Y}$ as $\hat{Y}=h\left(Y\right)$. Then, the Markov chain condition $\hat{Y}\to T\left(X\right)\to X$ does not hold. For this special case, Theorems 32.5 and 32.6 in [10] show that there a loss function $\hat{\ell }:{\left\{0,1\right\}}^{2}\to R_{+}$ exists, such that $L_{\hat{\ell }}^{*}\left(\hat{Y}|T\left(X\right)\right)>L_{\hat{\ell }}^{*}\left(\hat{Y}|X\right)$. Finally, letting $\ell \left(y,y^{\prime }\right)=\hat{\ell }\left(h\left(y\right),h\left(y^{\prime }\right)\right)$, we have

$$L_{\ell }^{*}\left(Y|T\left(X\right)\right)=L_{\hat{\ell }}^{*}\left(\hat{Y}|T\left(X\right)\right)>L_{\hat{\ell }}^{*}\left(\hat{Y}|X\right)=L_{\ell }^{*}\left(Y|X\right),$$

which shows that T(X) is not universally lossless. □

### 2.2. A Strongly Consistent Test

Theorem 1 implies an equivalent form of the losslessness null hypothesis defined by (1)

(2) $$\begin{array}{c} H_{0}=\left\{T:\;Y\to T\left(X\right)\to X\mathrm{is}\;a\;\mathrm{Markov}\;\mathrm{chain}\right\}, \end{array}$$

or equivalently, $H_{0}$ holds if and only if *X* and *Y* are conditionally independent given T(X):

$$H_{0}:P\left(X\in A,Y\in B|T(X)\right)=P\left(X\in A|T(X)\right)P\left(Y\in B|T(X)\right)\;a.s.$$

for arbitrary Borel sets A,B. Furthermore, we consider the general case where the alternative hypothesis $H_{1}$ is the complement of $H_{0}$: $H_{1}=H_{0}^{c}$.

Now, assume that the joint distribution of (X,Y,T(X)) is not known but instead a sample of independent and identically distributed (i.i.d.) random vectors $\left(X_{1},Y_{1},Z_{1}\right),\ldots ,\left(X_{n},Y_{n},Z_{n}\right)$ having a common distribution of (X,Y,Z) is given, where $Z_{i}=T\left(X_{i}\right)$ and Z=T(X).The goal is to test the hypothesis $H_{0}$ of conditional independence based on these data. In fact, our goal is to provide a strongly consistent test; i.e., a test that, with a probability of one, only makes finitely many Type I and II errors.

For testing conditional independence, most of the results in the literature used real valued X,Y,Z. Based on kernel density estimation, Cai et al. [11] introduced a test statistic and under the null hypothesis calculated its limit distribution. In Neykov et al. [12], a gap was introduced between the null and alternative hypotheses. This gap was characterized by the total variation distance, which decreased with increasing *n*. Under certain smoothness conditions, minimax bounds were derived. According to Shah and Peters [13], a regularity condition such as our Lipschitz condition (5) below cannot be omitted if a test for conditional independence is to be consistent. This is a consequence of their no-free lunch theorem that states that, under general conditions, if with the null hypothesis the bound on the error probability is non-asymptotic, then under the alternative hypothesis the rate of convergence of the error probability can be arbitrarily slow, which is a well-known phenomenon in nonparametric statistics. We note that these cited results, and indeed most of the results in the literature when testing for conditional independence, were for real-valued random variables and/or assumed certain special distribution types, typically the existence of a joint probability density function or that both *X* and *Y* are discrete, as in [12]. As we remarked earlier, such assumptions exclude problems where *Y* is discrete and *X* is continuous (typical in classification) or problems where the observation *X* is concentrated on a lower dimensional subspace or manifold. In contrast, our test construction is completely distribution-free and its convergence properties are almost distribution-free (we do assume a mild Lipschitz-type condition; see the upcoming Condition 1).

In our hypotheses testing setup, the alternative hypothesis, $H_{1}$, is the complement to the null hypothesis, $H_{0}$; therefore, there is no separation gap between the hypotheses. Dembo and Peres [14] and Nobel [15] characterized hypothesis pairs that admitted strongly consistent tests; i.e., tests that with a probability of one only make finitely many Type I and II errors. This property is called discernibility. As an illustration of the intricate nature of the discernibility concept, Dembo and Peres [14] demonstrated an exotic example, where the null hypothesis is that the mean of a random variable is rational, while the alternative hypothesis is that this mean minus $\sqrt{2}$ is rational. (See also Cover [16] and Kulkarni and Zeitouni [17].) The discernibility property shows up in Biau and Györfi [18] (testing homogeneity), Devroye and Lugosi [19] (classification of densities), Gretton and Györfi [20] (testing independence), Morvai and Weiss [21] and Nobel [15] (classification of stationary processes), among others.

In the remainder of this section, under mild conditions for the distribution of (X,Y), we study discernibility in the context of lossless transformations for statistical inference with general risk. We will make strong use of the multivariate-partitioning-based test of Györfi and Walk [2].

Let $P_{XYZ}$ denote the joint distribution of (X,Y,Z) and similarly for any marginal distribution of (X,Y,Z); e.g., $P_{XZ}$ denotes the distribution of the pair (X,Z). As in Györfi and Walk [2], introduce the following empirical distributions:

$$\begin{array}{cc} P_{XYZ}^{n}\left(A,B,C\right) & =\frac{\#\{i:\left(X_{i},Y_{i},Z_{i}\right)\in A\times B\times C,i=1,\ldots ,n\}}{n},\\ P_{XZ}^{n}\left(A,C\right) & =\frac{\#\{i:\left(X_{i},Z_{i}\right)\in A\times C,i=1,\ldots ,n\}}{n},\\ P_{YZ}^{n}\left(B,C\right) & =\frac{\#\{i:\left(Y_{i},Z_{i}\right)\in B\times C,i=1,\ldots ,n\}}{n}, \end{array}$$

and

$$P_{Z}^{n}\left(C\right)=\frac{\#\{i:Z_{i}\in C,i=1,\ldots ,n\}}{n},$$

for Borel sets $A\subset R^{d}$, B⊂R, and $C\subset R^{d^{\prime }}$.

For the sake of simplicity, assume that *X*, *Y*, and Z=T(X) are bounded. Otherwise, we apply a componentwise, one-to-one scaling in the interval [0,1]. Obviously, the losslessness null hypothesis $H_{0}$ is invariant under such a scaling. Let

$$P_{n}=\left\{A_{n,1},\ldots ,A_{n,m_{n}}\right\},\;Q_{n}=\left\{B_{n,1},\ldots ,B_{n,m_{n}^{\prime }}\right\},\;R_{n}=\left\{C_{n,1},\ldots ,C_{n,m_{n}^{\prime \prime }}\right\}$$

be the finite cubic partitions of the ranges *X*, *Y*, and *Z*, with all the cubes having common side lengths $h_{n}$ (thus, $h_{n}$ is proportional to $1/m_{n}^{\prime }$). As in [2], we define the test statistic

(3) $$\begin{array}{cc} L_{n}\; & =\sum_{A\in P_{n},B\in Q_{n},C\in R_{n}}\left|P_{XYZ}^{n}\left(A,B,C\right)-\frac{P_{XZ}^{n}\left(A,C\right)P_{YZ}^{n}\left(B,C\right)}{P_{Z}^{n}\left(C\right)}\right|. \end{array}$$

Our test rejects $H_{0}$ if

$$L_{n}\geq t_{n},$$

and accepts it if $L_{n}<t_{n}$, where the threshold $t_{n}$ is set to

(4) $$\begin{array}{cc} t_{n} & =c_{1}\left(\sqrt{\frac{m_{n}m_{n}^{\prime }m_{n}^{\prime \prime }}{n}}+\sqrt{\frac{m_{n}^{\prime }m_{n}^{\prime \prime }}{n}}+\sqrt{\frac{m_{n}m_{n}^{\prime \prime }}{n}}+\sqrt{\frac{m_{n}^{\prime \prime }}{n}}\right)+\left(\log n\right)h_{n}, \end{array}$$

where the constant $c_{1}$ satisfies

$$c_{1}>\sqrt{2\log2}\approx 1.177.$$

In this setup, the distribution of (X,Y) is arbitrary; its components can be discrete or absolutely continuous, or a mixture of the two or even singularly continuous. It is important to note that to construct this test, there is no need to know the type of distribution.

We assume that the joint distribution of *X*, *Y*, and Z=T(X) satisfies the following assumption.

**Condition** **1.** *Let p(·|z) be the density of the conditional distribution $P_{X|Z=z}=P\left(X\in \cdot \;|Z=z\right)$ with respect to the distribution $P_{X}$ as a dominating measure and introduce the notation*

$$C_{n}\left(z\right)=C_{n,j}ifz\in C_{n,j}.$$

*Assume that for some $C^{*}>0$, p(x|z) satisfies the condition*

(5) $$\begin{array}{cc} \int \int |p\left(x|z\right)-\frac{\int_{C_{n}\left(z\right)}p\left(x|z^{\prime }\right)P_{Z}\left(dz^{\prime }\right)}{P_{Z}\left(C_{n}\left(z\right)\right)}|\;P_{X}\left(dx\right)P_{Z}\left(dz\right)\; & \leq C^{*}h_{n}, \end{array}$$

*for all n.*

We note that the ordinary Lipschitz condition

(6) $$\begin{array}{cc} \int |p\left(x|z\right)-p\left(x|z^{\prime }\right)|\;P_{X}\left(dx\right)\; & \leq \frac{C^{*}}{\sqrt{d^{\prime }}}∥z-z^{\prime }∥\;\mathrm{for}\;\mathrm{all}\;z,z^{\prime }\in R^{d^{\prime }} \end{array}$$

implies (5). This latter condition is equivalent to

$$d_{TV}\left(P_{X|Z=z}\;,P_{X|Z=z^{\prime }}\right)\leq \frac{C^{*}}{2\sqrt{d^{\prime }}}∥z-z^{\prime }∥\;\mathrm{for}\;\mathrm{all}\;z,z^{\prime }\in R^{d^{\prime }},$$

where $d_{TV}\left(P,Q\right)$ denotes the total variation distance between distributions *P* and *Q*. In Neykov, Balakrishnan, and Wasserman [12], condition (6) is called the Null TV Lipschitz condition.

The next theorem is an adaptation and extension of the results in Györfi and Walk [2] to this particular problem of lossless transformation. In [2], it was assumed that the sequence of partitions $\left\{P_{n},Q_{n},R_{n}\right\}$ is nested, while we make no such assumption. The proof, in which an error made in [2] is also corrected, is relegated to Section 5.

**Theorem** **2.** *Suppose that X, Y, and Z=T(X) are bounded and Condition 1 holds for all n. If the sequence $h_{n}$ satisfies*

(7) $$\lim_{n\to \infty }nh_{n}^{d+1+d^{\prime }}=\infty$$

*and*

(8) $$\begin{array}{c} \lim_{n\to \infty }h_{n}^{d^{\prime }}\log n=0, \end{array}$$

*then we have the following:*

(a) *Under the losslessness null hypothesis $H_{0}$, we have for all $n\geq e^{C^{*}}$,*
(9) $$P\left(L_{n}\geq t_{n}\right)\leq 4e^{-\left(c_{1}^{2}/2-\log2\right)m_{n}^{\prime \prime }},$$
*and therefore, because $\sum_{n=1}^{\infty }P\left(L_{n}\geq t_{n}\right)<\infty$ by (8) and (9), after a random sample size, the test produces no error with a probability of one.*
(b) *Under the alternative hypothesis $H_{1}=H_{0}^{c}$,*
$$\underset{n\to \infty }{lim inf}L_{n}>0\;a.s.,$$
*thus, with a probability of one, after a random sample size, the test produces no error.*

**Remark** **1.**

(i) *The choice $h_{n}=n^{-\delta }$ with $0<\delta <1/(d+1+d^{\prime })$ satisfies both conditions (7) and (8).*
(ii) *Note that using (4), $t_{n}$ is of order $c_{1}\sqrt{\frac{m_{n}m_{n}^{\prime }m_{n}^{\prime \prime }}{n}}+\left(\log n\right)h_{n}$. Since we have*
$$\begin{array}{c} m_{n}=O\left(1/h_{n}^{d}\right),\;m_{n}^{\prime }=O\left(1/h_{n}\right),\;m_{n}^{\prime \prime }=O\left(1/h_{n}^{d^{\prime }}\right), \end{array}$$

*this means that $t_{n}$ is of order $\sqrt{1/(nh_{n}^{d+1+d^{\prime }})}+\left(\log n\right)h_{n}$.*

An important special transformation is given by the feature selection $X_{S}$ defined in the Introduction. Theorem 2 demonstrates the possibility of universally lossless dimension reduction for any multivariate feature vector. Note that in the setup of feature selection, the partition $P_{n}$ can be the nested version of $R_{n}$ and so the calculation of the test statistic $L_{n}$ is easier.

## 3. Universally δ-Lossless Transformations

Here, we develop bounds on the excess minimum risk, in terms of mutual information under various assumptions about the loss function. With the aid of these bounds, we give information-theoretic sufficient conditions for a transformation *T* to be universally δ-lossless with respect to fairly general classes of loss functions *ℓ*.

### 3.1. Preliminaries on Mutual Information

Let $P_{XY}$ denote the joint distribution of the pair (X,Y) and let $P_{X}P_{Y}$ denote the product of the marginal distributions of *X* and *Y*, respectively. The mutual information between *X* and *Y*, denoted by I(X;Y), is defined as

$$I\left(X;Y\right)=D\left(P_{XY}∥P_{X}P_{Y}\right),$$

where

$$D\left(P∥Q\right)=\left\{\begin{array}{cc} \int \frac{dP}{dQ}\log\left(\frac{dP}{dQ}\right)\;dQ & \;\mathrm{if}\;P\ll Q\\ \infty & \;\mathrm{otherwise}, \end{array}\right.$$

is the Kullback–Leibler (KL) divergence between probability distributions *P* and *Q* (here, P≪Q means that *P* is absolutely continuous with respect to *Q* with the Radon–Nikodym derivative $\frac{dP}{dQ}$). Thus, I(X;Y) is always nonnegative and I(X;Y)=0 if and only if *X* and *Y* are independent (note that I(X;Y)=∞ is possible). In this definition and throughout the paper, log denotes the natural logarithm.

For random variables *U* and *V* (both taking values in finite-dimensional Euclidean spaces), let $P_{U|V}$ denote the conditional distribution of *U*, given *V*. Furthermore, let $P_{U|V=v}$ denote the stochastic kernel (regular conditional probability) induced by $P_{U|V}$. Thus, in particular, $P_{U|V=v}\left(A\right)=P\left(U\in A|V=v\right)$ for each measurable set *A*.

Given another random variable *Z*, the conditional mutual information I(X;Y|Z) is defined as

$$I\left(X;Y|Z\right)=\int D\left(P_{XY|Z=z}∥P_{X|Z=z}P_{Y|Z=z}\right)P_{Z}\left(dz\right).$$

The integral above can also be denoted by $D(P_{YX|Z}∥P_{Y|Z}P_{X|Z}|P_{Z})$ and is called the conditional KL divergence. One can define

$$I\left(X;Y|Z=z\right)=D\left(P_{XY|Z=z}∥P_{X|Z=z}P_{Y|Z=z}\right)$$

so that

(10) $$I\left(X;Y|Z\right)=\int I\left(X;Y|Z=z\right)P_{Z}\left(dz\right).$$

From this definition it is clear that I(X;Y|Z)=0 if and only if *X* and *Y* are conditionally independent given *Z*, i.e., if and only if Y→Z→X (or equivalently, if and only if X→Z→Y).

Another way of expressing I(X;Y) is

(11) $$I\left(X;Y\right)=\int D\left(P_{Y|X=x}∥P_{Y}\right)P_{X}\left(dx\right).$$

One can see that in a similar way to I(X;Y|Z) can be expressed as

(12) $$I\left(X;Y|Z\right)=\;\int \int \;D\left(P_{Y|X=x,Z=z}∥P_{Y|Z=z}\right)P_{X|Z=z}\left(dx\right)P_{Z}\left(dz\right).$$

Properties of mutual information and conditional mutual information, their connections to the KL divergence, and identities involving these information measures are detailed in, e.g., Cover and Thomas ([22], Chapter 2) and Polyanskiy and Wu ([9], Chapter 3).

### 3.2. Mutual Information Bounds and δ-Lossless Transformations

A real random variable *U* with finite expectation is said to be $\sigma^{2}$-sub-Gaussian for some $\sigma^{2}>0$ if

$$\log E\left[e^{\lambda (U-E[U])}\right]\leq \frac{\sigma^{2}\lambda^{2}}{2}\;\mathrm{for}\;\mathrm{all}\;\lambda \in R.$$

Furthermore, we say that *U* is conditionally $\sigma^{2}$-sub-Gaussian given another random variable *V* if we have a.s.

(13) $$\log E\left[e^{\lambda (U-E[U\;|\;V])}\;|\;V\right]\leq \frac{\sigma^{2}\lambda^{2}}{2}\;\mathrm{for}\;\mathrm{all}\;\lambda \in R.$$

The following result gives a quantitative upper bound on the excess minimum risk $L_{\ell }^{*}\left(Y|T\left(X\right)\right)-L_{\ell }^{*}\left(Y|X\right)$ in terms of the mutual information difference I(Y;X)−I(Y;T(X)) under certain, not too restrictive, conditions. Note that $L_{\ell }^{*}\left(Y|T\left(X\right)\right)-L_{\ell }^{*}\left(Y|X\right)\geq 0$ always holds.

Given ϵ>0, we call an estimator $f^{\prime }:R^{d}\to Y$ϵ-optimal if $E\left[\ell \left(Y,f^{\prime }\left(X\right)\right)\right]<L_{\ell }^{*}\left(Y|X\right)+\epsilon$.

**Theorem** **3.** *Let $T:R^{d}\to R^{d^{\prime }}$ be a measurable transformation and assume that for any ϵ>0, there exists an ϵ-optimal estimator $f^{\prime }$ of Y from X, such that $\ell (y,f^{\prime }\left(X\right)))$ is conditionally $\sigma^{2}\left(y\right)$-sub-Gaussian given T(X) for every y∈Y, i.e.,*

(14) $$\log E\left[e^{\lambda \left(\ell \left(y,f^{\prime }\left(X\right)\right)-E\left[\ell \left(y,f^{\prime }\left(X\right)\right)\;|\;T\left(X\right)\right]\right)}|T\left(X\right)\right]\leq \frac{\sigma^{2}\left(y\right)\lambda^{2}}{2}\;a.s$$

*for all λ∈R and y∈R, where $\sigma^{2}:R\to R_{+}$ satisfies $E[\sigma^{2}\left(Y\right)]<\infty$. Then, one has*

(15) $$\begin{array}{cc} L_{\ell }^{*}\left(Y|T\left(X\right)\right)-L_{\ell }^{*}\left(Y|X\right)\; & \leq \sqrt{2E\left[\sigma^{2}\left(Y\right)\right]I\left(Y;X\;|\;T\left(X\right)\right)}\\ \; & =\sqrt{2E\left[\sigma^{2}\left(Y\right)\right]\left(I(Y;X)-I(Y;T(X))\right)}\;. \end{array}$$

**Remark** **2.**

(i) *In case I(Y;X|T(X))=∞, we interpret the right hand side of (15) as ∞. With this interpretation, the bound always holds.*
(ii) *We show in Section 4.2 that the sub-Gaussian condition (14) holds for the regression problem with squared error $\ell \left(y,y^{\prime }\right)={\left(y-y^{\prime }\right)}^{2}$ if Y=m(X)+N, where N is independent noise having a zero mean and finite fourth moment $E[N^{4}]<\infty$, and the regression function m(x)=E[Y|X=x] is bounded. In particular, the bound in the theorem holds if N is normal with zero mean and m is bounded.*
*We note that Theorem 6 and Corollary 3 in Xu and Raginsky [4] give bounds similar to (15), in the somewhat different context of Bayesian learning. However, the conditions there exclude, e.g., regression models in the form Y=m(X)+N if $E[|N{|}^{\delta }]=\infty$ for some δ>0.*
(iii) *Although hidden in the notation, $E[\sigma^{2}\left(Y\right)]$ depends on the loss function ℓ. Thus, the upper bound (15) is the product of two terms, the second of which,*
$$\sqrt{I(Y;X)-I(Y;T(X))},$$
*is independent of the loss function.*
(iv) *The bound in the theorem is not tight in general. In Section 4.3, an example is given in the context of portfolio selection, where the excess risk can be upper bounded by the difference I(Y;X)−I(Y;T(X)).*
(v) *The proof of Theorem 3 and those of its corollaries go through virtually without change if we replace T(X) with any $R^{d^{\prime }}$-valued random variable Z, such that Y→X→Z. Under the conditions of the theorem, we then have*
$$\begin{array}{cc} L_{\ell }^{*}\left(Y|Z\right)-L_{\ell }^{*}\left(Y|X\right)\; & \leq \sqrt{2E[\sigma^{2}\left(Y\right)]I\left(Y;X|Z\right)}\\ \; & =\sqrt{2E\left[\sigma^{2}\left(Y\right)\right]\left(I(Y;X)-I(Y;Z)\right)}\;. \end{array}$$
*In fact, Theorem 3 and its corollaries hold for general random variables Y, X, and Z taking values in complete and separable metric (Polish) spaces Y, X, and Z, respectively, if Y→X→Z.*

The proof of Theorem 3 is based on a slight generalization of Raginsky et al. ([5], Lemma 10.2), which we state next. In the lemma, *U* and *V* are arbitrary abstract random variables defined for the same probability space and taking values in spaces U and V, respectively; $\bar{U}$ and $\bar{V}$ are independent copies of *U* and *V* (so that $P_{\bar{U}\bar{V}}=P_{U}P_{V}$); and h:U×V→R is a measurable function.

**Lemma** **1.** *Assume that h(u,V) is $\sigma^{2}\left(u\right)$-sub-Gaussian for all u∈U, where $E[\sigma^{2}\left(U\right)]<\infty$. Then,*

$$|E\left[h\left(U,V\right)\right]-E\left[h\left(\bar{U},\bar{V}\right)\right]|\leq \sqrt{2E[\sigma^{2}\left(U\right)]I\left(U;V\right)}\;.$$

**Proof.**  We essentially copy the proof of ([5], Lemma 10.2), where it was assumed that $\sigma^{2}\left(u\right)$ does not depend on *u*. With this restriction, the sub-Gaussian condition (14) in Theorem 3 would have to hold with $\sigma^{2}\left(y\right)\leq \sigma^{2}$ uniformly over *y*. This condition would exclude regression models with independent sub-Gaussian noise and, *a fortiori*, models with independent noise that do not possess finite absolute moments of all orders, while our Theorem 2 can also be applied in such cases (see Section 4.2).

We make use of the Donsker–Varadhan variational representation of the relative entropy ([23] Corollary 4.15), which states that

$$D\left(P∥Q\right)=\underset{F}{\sup}\left(\;\int FdP-\log\int e^{F}dQ\;\right),$$

where the supremum is over all measurable F:Ω→R, such that $\int e^{F}dQ<\infty$. Applying this with $P=P_{V|U=u}$, $Q=P_{V}$, and F=λh(u,V), we obtain

(16) $$\begin{array}{cc} D(P_{V|U=u}∥P_{V})\; & \geq E\left[\lambda h\left(u,V\right)|U=u\right]-\log E\left[e^{\lambda h(u,V)}\right]\\ \; & \geq \lambda \left(E[h(u,V)|U=u]-E[\lambda h(u,V)]\right)-\frac{\lambda^{2}\sigma^{2}\left(u\right)}{2}, \end{array}$$

where the second inequality follows from the assumption that h(u,V) is $\sigma^{2}\left(u\right)$-sub-Gaussian. Maximizing the right-hand side of (16) over λ∈R gives, after rearrangement,

(17) $$|E\left[h\left(u,V\right)|U=u\right]-E\left[h\left(u,V\right)\right]|\leq \sqrt{2\sigma^{2}\left(u\right)D\left(P_{V|U=u}∥P_{V}\right)}.$$

Since $\bar{U}$ and $\bar{V}$ are independent, $E\left[h\left(u,V\right)\right]=E\left[h\left(\bar{U},\bar{V}\right)|\bar{U}=u\right]$, and we obtain

(18) $$\begin{array}{l} \left|E\left[h\left(U,V\right)\right]-E\left[h\left(\bar{U},\bar{V}\right)\right]\right|\\ =|\int \left(E\left[h\left(U,V\right)|U=u\right]-E\left[h\left(\bar{U},\bar{V}\right)|\bar{U}=u\right]\right)\;P_{U}\left(du\right)|\\ =|\int \left(E[h(u,V)|U=u]-h(u,V)\right)\;P_{U}\left(du\right)|\\ \leq \int |E\left[h\left(u,V\right)|U=u\right]-h\left(u,V\right)|\;P_{U}\left(du\right) \end{array}$$

(19) $$\leq \int \sqrt{2\sigma^{2}\left(u\right)D\left(P_{V|U=u}∥P_{V}\right)}\;P_{U}\left(du\right)$$

(20) $$\leq \sqrt{\int 2\sigma^{2}\left(u\right)\;P_{U}\left(du\right)}\;\sqrt{\int D\left(P_{V|U=u}∥P_{V}\right)\;P_{U}\left(du\right)}$$

(21) $$=\sqrt{2E[\sigma^{2}\left(U\right)]I\left(U;V\right)},$$

where (18) follows from Jensen’s inequality, (19) follows from (17), in (20) we used the Cauchy–Schwarz inequality, and the last equality follows from (11). □

**Proof of Theorem** **3.** Let $\bar{Y}$ and $\bar{X}$ be random variables, such that $P_{\bar{Y}|T\left(\bar{X}\right)}=P_{Y|T(X)}$, $P_{\bar{X}|T\left(\bar{X}\right)}=P_{X|T(X)}$, $P_{T(\bar{X})}=P_{T(X)}$, and $\bar{Y}$ and $\bar{X}$ are conditionally independent given $T(\bar{X})$. Thus, the joint distribution of the triple $\left(\bar{Y},\bar{X},T\left(\bar{X}\right)\right)$ is $P_{\bar{Y}\bar{X}T\left(\bar{X}\right)}=P_{Y|T(X)}P_{X|T(X)}P_{T(X)}$.

We apply Lemma 1 with U=Y, V=X, and $h\left(u,v\right)=\ell (y,f^{\prime }\left(x\right))$. Note that, using the conditions of the theorem, we can choose an ϵ-optimal $f^{\prime }$, such that for every *y*, $\ell (y,f^{\prime }\left(X\right))$ is conditionally $\sigma^{2}\left(y\right)$-sub-Gaussian given T(X). Consider $E[\ell \left(Y,f^{\prime }\left(X\right)\right)\;|\;T\left(X\right)=z]$ and $E[\ell \left(\bar{Y},f^{\prime }\left(\bar{X}\right)\right),|\;T\left(\bar{X}\right)=z]$ as regular (unconditional) expectations taken with respect to $P_{YX|T(X)=z}$ and $P_{\bar{Y}\bar{X}|T\left(\bar{X}\right)=z}$ respectively, and consider I(Y;X|T(X)=z) as regular mutual information between random variables with the distribution $P_{YX|T(X)=z}$. Since $\bar{Y}$ and $\bar{X}$ are conditionally independent given $T(\bar{X})=z$, Lemma 1 yields

$$\begin{array}{c} \left|E\left[\ell \left(Y,f^{\prime }\left(X\right)\right)\;|\;T\left(X\right)=z\right]-E\left[\ell \left(\bar{Y},f^{\prime }\left(\bar{Y}\right)\right)\;|\;T\left(\bar{X}\right)=z\right]\right|\\ \leq \sqrt{2E\left[\sigma^{2}\left(Y\right)|T\left(X\right)=z\right]I\left(X;Y|T\left(X\right)=z\right)}\;. \end{array}$$

Recalling that $T(\bar{X})$ and T(X) have the same distribution, and applying Jensen’s inequality and the Cauchy–Schwarz inequality as in (18) and (20), we obtain

(22) $$\begin{array}{c} \left|E\left[\ell \left(Y,f^{\prime }\left(X\right)\right)\right]-E\left[\ell \left(\bar{Y},f^{\prime }\left(\bar{X}\right)\right)\right]\right|\\ \leq \int |E\left[\ell \left(Y,f^{\prime }\left(X\right)\right)\;|\;T\left(X\right)=z\right]-E\left[\ell \left(\bar{Y},f^{\prime }\left(\bar{X}\right)\right)\;|\;T\left(\bar{X}\right)=z\right]|\;P_{T(X)}\left(dz\right)\\ \leq \int \sqrt{2E\left[\sigma^{2}\left(Y\right)|T\left(X\right)=z\right]I\left(Y;X|T\left(X\right)=z\right)}\;P_{T(X)}\left(dz\right)\\ \leq \sqrt{2\int E\left[\sigma^{2}\left(Y\right)|T\left(X\right)=z\right]\;P_{T(X)}\left(dz\right)}\;\sqrt{\int I\left(Y;X|T\left(X\right)=z\right)\;P_{T(X)}\left(dz\right)}\\ =\sqrt{2E\left[\sigma^{2}\left(Y\right)\right]I\left(Y;X|T\left(X\right)\right)}\;. \end{array}$$

On the one hand, we have

(23) $$E\left[\ell (\bar{Y};f^{\prime }\left(\bar{X}\right))\right]\geq L_{\ell }^{*}\left(\bar{Y}|\bar{X}\right)=L_{\ell }^{*}\left(\bar{Y}|T\left(\bar{X}\right)\right)=L_{\ell }^{*}\left(Y|T\left(X\right)\right),$$

where the first equality follows from Theorem 1 with the conditional independence of $\bar{Y}$ and $\bar{X}$ given $T(\bar{X})$, and the second follows, since $\left(\bar{Y},T\left(\bar{X}\right)\right)$ and (Y,T(X)) have the same distribution by construction. On the other hand, $L_{\ell }^{*}\left(Y|X\right)\geq E\left[\ell \left(Y,f^{\prime }\left(X\right)\right)\right]-\epsilon$. Thus, (22) and (23) imply

$$\begin{array}{cc} 0\leq L_{\ell }^{*}\left(Y|T\left(X\right)\right)-L_{\ell }^{*}\left(Y|X\right)\; & \leq E\left[\ell \left(\bar{Y},f^{\prime }\left(\bar{X}\right)\right)\right]-E\left[\ell \left(Y,f^{\prime }\left(X\right)\right)\right]+\epsilon \\ \; & \leq \sqrt{2E\left[\sigma^{2}\left(Y\right)\right]I\left(Y;X|T\left(X\right)\right)}+\epsilon , \end{array}$$

which proves the upper bound in (15), since ϵ>0 is arbitrary. By expanding I(Y;X|Z) in two different ways using the chain rule for mutual information (e.g., Cover and Thomas ([22], Thm. 2.5.2)), and using the conditional independence of *Y* and T(X) given *X*, one obtains I(Y;X|T(X))=I(Y;X)−I(Y;T(X)), which shows the equality in (15). □

We state two corollaries for special cases. In the first, we assume that *ℓ* is uniformly bounded, i.e., ${∥\ell ∥}_{\infty }=\sup_{y,y^{\prime }\in Y}\ell \left(y,y^{\prime }\right)<\infty$. For any c>0, let L(c) denote the collection of all loss functions *ℓ* with ${∥\ell ∥}_{\infty }\leq c$. Recall the notion of a universally δ-lossless transformation from Definition 2.

**Corollary** **1.** *Suppose the loss function ℓ is bounded. Then, for any measurable $T:R^{d}\to R^{d^{\prime }}$, we have*

(24) $$\begin{array}{cc} L_{\ell }^{*}\left(Y|T\left(X\right)\right)-L_{\ell }^{*}\left(Y|X\right) & \leq \frac{{∥\ell ∥}_{\infty }}{\sqrt{2}}\sqrt{I(Y;X)-I(Y;T(X))}\;. \end{array}$$

*Therefore, whenever*

(25) $$I\left(Y;X\right)-I\left(Y;T\left(X\right)\right)\leq \frac{2\delta^{2}}{c^{2}},$$

*the transformation T is universally δ-lossless for the family L(c), i.e., $L_{\ell }^{*}\left(Y|T\left(X\right)\right)-L_{\ell }^{*}\left(Y|X\right)\leq \delta$ for all ℓ with ${∥\ell ∥}_{\infty }\leq c$.*

**Remark** **3.**

(i) *The bound of the theorem can be used to give an estimation-theoretic motivation of the information bottleneck (IB) problem; see Section 4.4.*
(ii) *Let $L_{\ell }^{*}\left(Y\right)=L_{\ell }^{*}\left(Y|\emptyset \right)=\inf_{y\in Y}E\left[\ell \left(Y,y\right)\right]$. For bounded ℓ, the inequality*
$$L_{\ell }^{*}\left(Y\right)-L_{\ell }^{*}\left(Y|X\right)\leq 2\sqrt{2}{∥\ell ∥}_{\infty }\sqrt{I(Y;X)}$$
*was proven in Makhdoumi et al. ([24], Theorem 1) for discrete alphabets, to solve the so-called privacy funnel problem.This inequality follows from (15) by setting Z=T(X) to be constant there.*
(iii) *A simple self-contained proof of (24) (see below) was provided by Or Ordentlich and communicated to the second author by Shlomo Shamai [25], in response to an early version of this manuscript. The bound in (24) seems to have first appeared in published form in Hafez-Kolahi et al. ([26], Lemma 1), where the proof was attributed to Xu and Raginsky [27].*

**Proof of Corollary** **1.**  If *ℓ* is uniformly bounded, then for any $f:R^{d}\to Y$ one has $\ell \left(y,f\left(x\right)\right)\in [0,∥\ell {∥}_{\infty }]$ for all *y* and *x*. Then Hoeffding’s lemma (e.g., Boucheron et al. ([23], Lemma 2.2)) implies that for all *y*, ℓ(y,f(X)) is conditionally $\sigma^{2}$-sub-Gaussian with $\sigma^{2}=\frac{{∥\ell ∥}_{\infty }^{2}}{4}$ given T(X). Since an ϵ-optimal estimator $f^{\prime }$ exists for any ϵ>0 and $\ell (y,f^{\prime }\left(X\right))$ is conditionally $\sigma^{2}$-sub-Gaussian, given T(X) using the preceding argument, (24) follows from Theorem 3. The second statement follows directly from (24) and the fact that ${∥\ell ∥}_{\infty }\leq c$ for all ℓ∈L(c).

The following alternative argument by Or Ordentlich [25] is based on Pinsker’s inequality for the total variation distance in terms of the KL divergence (see, e.g., ([9], Theorem 7.9)). For bounded *ℓ*, this gives a direct proof of an analogue of the key inequality (22) in the proof of Theorem 3. This argument avoids Lemma 1 and the machinery introduced by the sub-Gaussian assumption.

Using the same notation as in the proof of Theorem 3 and letting $P=P_{YXZ}$ and $Q=P_{\bar{Y}\bar{X}T\left(\bar{Y}\right)}$, we have

$$\begin{array}{cc} E\left[\ell (Y,f^{\prime }\left(X\right))\right]-E\left[\ell (\bar{Y},f^{\prime }\left(\bar{X}\right))\right]| & =\int \ell \left(y,f^{\prime }\left(x\right)\right)\;dP-\int \ell \left(y,f^{\prime }\left(x\right)\right)\;dQ\\ \; & \leq {∥\ell ∥}_{\infty }d_{\mathrm{TV}}\left(P,Q\right)\\ \; & \leq \frac{{∥\ell ∥}_{\infty }}{\sqrt{2}}\sqrt{D(P∥Q)}\;(\mathrm{by}\;\mathrm{Pinsker}\prime s\;\mathrm{inequality})\\ \; & =\frac{{∥\ell ∥}_{\infty }}{\sqrt{2}}\sqrt{D(P_{YX|T(X)}P_{Z}∥P_{Y|T(X)}P_{X|T(X)}P_{T(X)})}\\ \; & =\frac{{∥\ell ∥}_{\infty }}{\sqrt{2}}\sqrt{D(P_{YX|T(X)}∥P_{Y|T(X)}P_{X|T(X)}|P_{T(X)})}\\ \; & =\frac{{∥\ell ∥}_{\infty }}{\sqrt{2}}\sqrt{I(X,Y|T(X))}\;. \end{array}$$

The rest of the proof proceeds exactly as in Theorem 3. □

In the second corollary, we do not require that *ℓ* be bounded but assume that an optimal estimator $f_{\ell }^{*}$ from *X* to *Y* exists, such that $\ell (y,f_{\ell }^{*}\left(X\right))$ is conditionally $\sigma^{2}\left(y\right)$-sub-Gaussian given T(X), where $E[\sigma^{2}\left(Y\right)]<\infty$.

**Corollary** **2.** *Assume that an optimal estimator $f_{\ell }^{*}$ of Y from X exists, i.e., the measurable function $f_{\ell }^{*}$ satisfies $E\left[\ell \left(Y,f_{\ell }^{*}\left(X\right)\right)\right]=L_{\ell }^{*}\left(Y|X\right)$. Furthermore, suppose that the sub-Gaussian condition of Theorem 3 holds with $f^{\prime }=f_{\ell }^{*}$ (i.e., (14) holds for $f^{\prime }=f_{\ell }^{*})$. Then,*

(26) $$\begin{array}{cc} L_{\ell }^{*}\left(Y|T\left(X\right)\right)-L_{\ell }^{*}\left(Y|X\right) & \leq \sqrt{2E\left[\sigma^{2}\left(Y\right)\right]\left(I(Y;X)-I(Y;T(X))\right)}\;. \end{array}$$

**Proof.**  The corollary immediately follows from Theorem 3, since an optimal $f_{\ell }^{*}$ is ϵ-optimal for all ϵ>0. □

For the next corollary, let $\hat{L}\left(c\right)$ denote the collection of all loss functions *ℓ*, such that

$$\ell \left(y,f_{\ell }^{*}\left(X\right)\right)\leq g_{\ell }\left(y\right)\;a.s.$$

for some function $g_{\ell }:Y\to R_{+}$ with $E\left[g_{\ell }^{2}\left(Y\right)\right]\leq c^{2}$.

**Corollary** **3.** *If T is a transformation such that*

$$I\left(Y;X\right)-I\left(Y;T\left(X\right)\right)\leq \frac{2\delta^{2}}{c^{2}},$$

*then T is universally δ-lossless for the family $\hat{L}\left(c\right)$.*

**Proof.**  Since $\ell (y,f_{\ell }^{*}\left(X\right))$ is a.s. upper bounded by $g_{\ell }\left(y\right)$ for any $\ell \in \hat{L}\left(c\right)$, using Hoeffding’s lemma ([23], Lemma 2.2), we have that $\ell (y,f_{\ell }^{*}\left(X\right))$ is conditionally $\frac{g_{\ell }^{2}\left(y\right)}{4}$-sub-Gaussian given T(X). Thus, from Corollary 2, for all $\ell \in \hat{L}\left(c\right)$, we have

$$\begin{array}{cc} L_{\ell }^{*}\left(Y|T\left(X\right)\right)-L_{\ell }^{*}\left(Y|X\right) & \leq \sqrt{\frac{1}{2}E\left[g_{\ell }^{2}\left(Y\right)\right]\left(I(Y;X)-I(Y;T(X))\right)}\\ \; & \leq \sqrt{\frac{c^{2}}{2}\left(I(Y;X)-I(Y;T(X))\right)}\\ \; & \leq \delta \end{array}$$

if $I\left(Y;X\right)-I\left(Y;T\left(X\right)\right)\leq \frac{2\delta^{2}}{c^{2}}$. □

The next corollary generalizes and gives a much simplified proof of Faragó and Györfi [28], see also Devroye, Györfi, and Lugosi ([10], Theorem. 32.3). This result states for binary classification (*Y* is 0-1-valued and $\ell \left(y,y^{\prime }\right)=I_{y\neq y^{\prime }}\;$) that if a sequence of functions $T_{n}:R^{d}\to R^{d}$ is such that $∥X-T_{n}\left(X\right)∥\to 0$ in probability as n→∞, then $L_{\ell }^{*}\left(Y|T_{n}\left(X\right)\right)\to L_{\ell }^{*}\left(Y|X\right)$ as n→∞.

**Corollary** **4.** *Assume that a sequence of transformations $T_{n}:R^{d}\to R^{d}$ is such that $T_{n}\left(X\right)\to X$ in distribution (i.e., $P_{T_{n}\left(X\right)}\to P_{X}$ weakly) as n→∞. Then, for any bounded loss function ℓ,*

(27) $$\lim_{n\to \infty }L_{\ell }^{*}\left(Y|T_{n}\left(X\right)\right)=L_{\ell }^{*}\left(Y|X\right).$$

Note that this corollary and its proof still hold without any changes if *X* takes values in an arbitrary complete separable metric space. For example, in the setup of function classification, *X* may take values in an $L_{p}$ function space for 1≤p<∞, and $T_{n}$ is a truncated series expansion or a quantizer. Interestingly, here the asymptotic losslessness property is guaranteed, even in the case where the sequence of transformations $T_{n}$ and the loss function *ℓ* are not matched at all.

**Proof.**  If $T_{n}\left(X\right)\to X$ in distribution, then clearly $(Y,T_{n}\left(X\right))\to \left(Y,X\right)$ in distribution. Thus, the lower semicontinuity of mutual information with respect to convergence in distribution (see, e.g., Polyanskiy and Wu ([9], Equation (4.28))) implies

$$\underset{n\to \infty }{lim inf}I\left(Y;T_{n}\left(X\right)\right)\geq I\left(Y;X\right).$$

Since $I(Y;T_{n}\left(X\right))\leq I\left(Y;X\right)$ for all *n*, we obtain

$$\lim_{n\to \infty }I\left(Y;T_{n}\left(X\right)\right)=I\left(Y;X\right).$$

Combined with Corollary 1 (with *T* replaced with $T_{n}$), this gives

$$\begin{array}{cc} 0\leq \lim_{n\to \infty }L_{\ell }^{*}\left(Y|T_{n}\left(X\right)\right)-L_{\ell }^{*}\left(Y|X\right) & \leq \lim_{n\to \infty }\frac{{∥\ell ∥}_{\infty }}{\sqrt{2}}\sqrt{I\left(Y;X\right)-I(Y;T_{n}\left(X\right))}\\ \; & =0. \end{array}$$

□

## 4. Applications

### 4.1. Classification

For classification, Y is the finite set {1,⋯,M} and the cost is the 0−1 loss

$$\begin{array}{c} \ell \left(y,y^{\prime }\right)=I_{y\neq y^{\prime }}. \end{array}$$

In this setup, the risk of estimator *f* is the error probability P(Y≠f(X)). With the notation

$$\begin{array}{c} P_{y}\left(x\right)=P\left(Y=y|X=x\right), \end{array}$$

the optimal estimator is the Bayes decision

$$\begin{array}{c} f^{*}\left(x\right)=\underset{y\in Y}{\arg\;\max}P_{y}\left(x\right), \end{array}$$

and the minimum risk is the Bayes error probability

$$\begin{array}{c} L^{*}\left(X\right)=1-E\left[\max_{y\in Y}P_{y}\left(X\right)\right]. \end{array}$$

If $L^{*}\left(T\left(X\right)\right)=1-E\left[\max_{y}E\left[P_{y}\left(X\right)|T\left(X\right)\right]\right]$ stands for the Bayes error probability of the transformed observation vector T(X), then (24) with ${∥\ell ∥}_{\infty }=1$ yields the upper bound

$$L^{*}\left(T\left(X\right)\right)-L^{*}\left(X\right)\leq \frac{1}{\sqrt{2}}\sqrt{I(Y;X)-I(Y;T(X))};$$

see also ([4], Corollary 2) for a similar bound in the context of Bayesian learning.

As a special case, the feature selector $X\mapsto X_{S}$ is lossless if

(28) $$\begin{array}{c} L^{*}\left(X\right)=L^{*}\left(X_{S}\right). \end{array}$$

Györfi and Walk [29] studied the corresponding hypothesis testing problem. Using a *k*-nearest-neighbor (*k*-NN) estimate of the excess Bayes error probability $L^{*}\left(X_{S}\right)-L^{*}\left(X\right)$, they introduced a test statistic and accepted the hypothesis (28), if the test statistic is less than a threshold. Under certain mild conditions, the strong consistency of this test has been proven.

### 4.2. Nonparametric Regression

For the nonparametric regression problem, the cost is the squared loss

$$\begin{array}{c} \ell \left(y,y^{\prime }\right)={\left(y-y^{\prime }\right)}^{2},\;y,y^{\prime }\in R, \end{array}$$

and the best statistical inference is the regression function

$$\begin{array}{c} m(X)=E[Y|X] \end{array}$$

(here, we assume $E[Y^{2}]<\infty$). Then, the minimum risk is the residual variance

$$\begin{array}{c} L_{\ell }^{*}\left(Y|X\right)=E\left[{\left(Y-m\left(X\right)\right)}^{2}\right]. \end{array}$$

If $L^{*}\left(X\right)=L_{\ell }^{*}\left(Y|X\right)$ and $L^{*}\left(T\left(X\right)\right)=L_{\ell }^{*}\left(Y|T\left(X\right)\right)$ denote the residual variances for the observation vectors *X* and T(X), respectively, then

$$\begin{array}{cc} L^{*}\left(T\left(X\right)\right)-L^{*}\left(X\right)\; & =E\left[{\left(Y-E\left[m\left(X\right)|T\left(X\right)\right]\right)}^{2}\right]-E\left[{\left(Y-m\left(X\right)\right)}^{2}\right]\\ \; & =E\left[{\left(m\left(X\right)-E\left[m\left(X\right)|T\left(X\right)\right]\right)}^{2}\right]. \end{array}$$

Note that the excess residual variance $L^{*}\left(T\left(X\right)\right)-L^{*}\left(X\right)$ does not depend on the distribution of the residual Y−m(X).

Next, we show that the conditions of Corollary 2 hold with $f_{\ell }^{*}\left(x\right)=m\left(x\right)$ for the important case

Y=m(X)+N,

where *N* is a zero-mean noise variable that is independent of *X* and satisfies $E[N^{4}]<\infty$, and *m* is bounded as |m(x)|≤K for all *x*. For this model, we have

$$\ell \left(y,f^{*}\left(X\right)\right)={\left(y-m\left(X\right)\right)}^{2}\leq \left(|y\right|+{\left|m\left(X\right)|\right)}^{2}\leq {\left(|y|+K\right)}^{2}.$$

Thus, $\ell (y,f^{*}\left(X\right))$ is a nonnegative random variable a.s. bounded by ${\left(|y|+K\right)}^{2}$, which implies via Hoeffding’s lemma (e.g., ([23], Lemma 2.2)) that it is $\sigma^{2}\left(y\right)$-sub-Gaussian given T(X) with $\sigma^{2}\left(y\right)=\frac{{\left(|y|+K\right)}^{4}}{4}$. We have

$$\begin{array}{cc} E[\sigma^{2}\left(Y\right)]\; & =\frac{E\left[{\left(|Y|+K\right)}^{4}\right]}{4}\\ \; & \leq \frac{E\left[{\left(|N|+|m\left(X\right)|+K\right)}^{4}\right]}{4}\\ \; & \leq \frac{E\left[{\left(|N|+2K\right)}^{4}\right]}{4}\\ \; & \leq \frac{8E\left[{\left|N\right|}^{4}+{\left(2K\right)}^{4}\right]}{4}\\ \; & \leq 2E\left[N^{4}\right]+32K^{4}\\ \; & <\infty , \end{array}$$

thus, the conditions of Corollary 2 hold and we obtain

$$\begin{array}{cc} L_{\ell }^{*}\left(Y|T\left(X\right)\right)-L_{\ell }^{*}\left(Y|X\right)\; & =E\left[{\left(Y-E\left[Y|T\left(X\right)\right]\right)}^{2}\right]-E\left[{\left(Y-E\left[Y|X\right]\right)}^{2}\right]\\ \; & \leq \sqrt{\left(2E\left[N^{4}\right]+32K^{4}\right)\left(I(Y;X)-I(Y;T(X))\right)}\;. \end{array}$$

Again, the feature selection $X_{S}$ is called lossless, when $L^{*}\left(X\right)=L^{*}\left(X_{S}\right)$ holds. As a test statistic, Devroye et al. [30] introduced a 1-NN estimate of $L^{*}\left(X_{S}\right)-L^{*}\left(X\right)$ and proved the strong consistency of the corresponding test.

### 4.3. Portfolio Selection

The next example is related to the negative of the log-loss or log-utility; see Algoet and Cover [31], Barron and Cover [32], Chapters 6 and 16 in Cover and Thomas [22], Györfi et al. [33].

Consider a market consisting of $d_{a}$ assets. The evolution of the market in time is represented by a sequence of (random) price vectors $S_{1},S_{2},\ldots \in R_{+}^{d_{a}}$ with

$$S_{n}=\left(S_{n}^{\left(1\right)},\cdots ,S_{n}^{\left(d_{a}\right)}\right),$$

where the *j*th component $S_{n}^{\left(j\right)}$ of $S_{n}$ denotes the price of the *j*th asset in the *n*th trading period. Let us transform the sequence of price vectors $\left\{S_{n}\right\}$ into the sequence of return (relative price) vectors $\left\{R_{n}\right\}$, defined as

$$R_{n}=\left(R_{n}^{\left(1\right)},\cdots ,R_{n}^{\left(d_{a}\right)}\right),$$

where

$$R_{n}^{\left(j\right)}=\frac{S_{n}^{\left(j\right)}}{S_{n-1}^{\left(j\right)}}.$$

Constantly rebalanced portfolio selection is a multi-period investment strategy, where at the beginning of each trading period the investor redistributes the wealth among the assets. The investor is allowed to diversify their capital at the beginning of each trading period according to a portfolio vector $b=(b^{\left(1\right)},\cdots b^{\left(d_{a}\right)})$. The *j*th component $b^{\left(j\right)}$ of *b* denotes the proportion of the investor’s capital invested in asset *j*. Here, we assume that the portfolio vector *b* has nonnegative components with $\sum_{j=1}^{d_{a}}b^{\left(j\right)}=1$. The simplex of possible portfolio vectors is denoted by $\Delta_{d_{a}}$.

Let $S_{0}=1$ denote the investor’s initial capital. Then, at the beginning of the first trading period, $S_{0}b^{\left(j\right)}$ is invested into asset *j*, and this results in return $S_{0}b^{\left(j\right)}R_{1}^{\left(j\right)}$, and therefore at the end of the first trading period the investor’s wealth becomes

$$S_{1}=S_{0}\sum_{j=1}^{d_{a}}b^{\left(j\right)}R_{1}^{\left(j\right)}=\langle b\;,\;R_{1}\rangle ,$$

where $\langle \;\cdot \;\;,\;\;\cdot \;\rangle$ denotes the standard inner product in $R^{d_{a}}$. For the second trading period, $S_{1}$ is the new initial capital

$$S_{2}=S_{1}\cdot \langle b\;,\;R_{2}\rangle =\langle b\;,\;R_{1}\rangle \cdot \langle b\;,\;R_{2}\rangle .$$

By induction, for the trading period *n*, the initial capital is $S_{n-1}$, and therefore

$$S_{n}=S_{n-1}\langle b\;,\;R_{n}\rangle =\prod_{i=1}^{n}\langle b\;,\;R_{i}\rangle .$$

The asymptotic average growth rate of this portfolio selection strategy is

$$\begin{array}{cc} \lim_{n\to \infty }\frac{1}{n}\log S_{n} & =\lim_{n\to \infty }\frac{1}{n}\sum_{i=1}^{n}\log\langle b\;,\;R_{i}\rangle \end{array}$$

assuming a limit exists.

If the market process $\left\{R_{i}\right\}$ is memory-less, i.e., it is a sequence of i.i.d. random return vectors, then the strong law of large numbers implies that the best constantly rebalanced portfolio (BCRP) is the log-optimal portfolio:

$$b^{*}=\underset{b\in \Delta_{d_{a}}}{\arg\;\max}E\left[\log\langle b\;,\;R_{1}\rangle \right],$$

while the best asymptotic average growth rate is

$$W^{*}=\max_{b\in \Delta_{d_{a}}}E\left[\log\langle b\;,\;R_{1}\rangle \right].$$

Barron and Cover [32] extended this setup to portfolio selection with side information. Assume that $X_{1},X_{2},\ldots$ are $R^{d}$ valued side information vectors, such that $\left(R_{1},X_{1}\right),\left(R_{2},X_{2}\right),\ldots$ are i.i.d. and in each round *n* the portfolio vector may depend on $X_{n}$. The strong law of large numbers yields

$$\begin{array}{cc} \lim_{n\to \infty }\frac{1}{n}\log S_{n} & =\lim_{n\to \infty }\frac{1}{n}\sum_{i=1}^{n}\log\langle b(X_{i})\;,\;R_{i}\rangle =E\left[\log\langle b(X_{1})\;,\;R_{1}\rangle \right]\;a.s. \end{array}$$

Therefore, the log-optimal portfolio has the form

$$b^{*}\left(X_{1}\right)=\underset{b\in \Delta_{d_{a}}}{\arg\;\max}E\left[\log\langle b\;,\;R_{1}\rangle |X_{1}\right]$$

and the best asymptotic average growth rate is

$$W^{*}\left(X\right)=E\left[\max_{b\in \Delta_{d_{a}}}E\left[\log\langle b\;,\;R_{1}\rangle |X_{1}\right]\right].$$

Barron and Cover ([32], Thm. 2) proved that

(29) $$W^{*}\left(X\right)-W^{*}\leq I\left(R_{1};X_{1}\right).$$

The next theorem generalizes this result by upper bounding the loss of the best asymptotic growth rate when, instead of *X*, only degraded side information T(X) is available.

**Theorem** **4.** *For any measurable $T:R^{d}\to R^{d^{\prime }}$,*

$$W^{*}\left(X\right)-W^{*}\left(T\left(X\right)\right)\leq I\left(R_{1};X_{1}\right)-I\left(R_{1};T\left(X_{1}\right)\right)$$

*assuming the terms on the right hand side are finite.*

**Remark** **4.**

(i) *As in Theorem 3, the difference $I\left(R_{1};X_{1}\right)-I\left(R_{1};T\left(X_{1}\right)\right)$ in the upper bound is equal to $I(R_{1};X_{1}|T\left(X_{1}\right))$, a quantity that is always nonnegative but may be equal to ∞. In this case, we interpret the right hand side as ∞.*
(ii) *There is a correspondence between this setup of portfolio selection and the setup in previous sections. In particular, Y from the previous sections is equal to R with a range $R_{+}^{d_{a}}$ and the inference is b(X) taking values in $\Delta_{d_{a}}$. Then, the loss is $-\log\langle b(X)\;,\;R\rangle$. If we assume that for all $j=1,\cdots d_{a}$,*
(30) $$\begin{array}{c} |\log R^{\left(j\right)}|\leq c_{max}\;a.s., \end{array}$$
*then*
$$\begin{array}{c} |\log\langle b(X)\;,\;R\rangle |\leq c_{max}\;a.s. \end{array}$$
*and so Corollary 1 implies*
$$W^{*}\left(X\right)-W^{*}\left(T\left(X\right)\right)\leq \frac{c_{max}}{\sqrt{2}}\sqrt{I\left(R_{1};X_{1}\right)-I\left(R_{1};T\left(X_{1}\right)\right)}.$$
*Note that, from the point of view of application, (30) is a mild condition. For example, for NYSE daily data $c_{max}\leq 0.3$; see Györfi et al. [34].*

**Proof.**  Let (R,X) be a generic copy of the $\left(R_{i},X_{i}\right)$. Writing out explicitly the dependence of $W^{*}$ on $P_{\;R}$, we have

$$W^{*}\left(X\right)=\int W^{*}\left(P_{\;R|X=x}\right)P_{X}\left(dx\right)$$

and from (11) we have

$$I\left(R;X\right)=\int D\left(P_{\;R|X=x}∥P_{\;R}\right)P_{X}\left(dx\right).$$

Thus, the bound $W^{*}\left(X\right)-W^{*}\leq I\left(R_{1};X_{1}\right)$ in (29) can be written as

(31) $$\begin{array}{cc} \int W^{*}\left(P_{\;R|X=x}\right)P_{X}\left(dx\right)-W^{*} & \leq \int D\left(P_{\;R|X=x}∥P_{\;R}\right)P_{X}\left(dx\right). \end{array}$$

Furthermore, letting Z=T(X), we have

$$\begin{array}{cc} W^{*}\left(X\right)-W^{*}\left(Z\right) & =\int W^{*}\left(P_{\;R|X=x}\right)P_{X}\left(dx\right)-\int W^{*}\left(P_{\;R|Z=z}\right)P_{Z}\left(dz\right). \end{array}$$

Since R→X→Z is a Markov chain, $P_{\;R|X=x}=P_{\;R|X=x,Z=z}$, and we obtain

$$\begin{array}{cc} W^{*}\left(X\right)-W^{*}\left(Z\right)\\ \; & =\int \left(\int W^{*}\left(P_{\;R|X=x,Z=z}\right)P_{X|Z=z}\left(dx\right)-W^{*}\left(P_{\;R|Z=z}\right)\right)P_{Z}\left(dz\right). \end{array}$$

Applying (31) with $W^{*}\left(P_{\;R|X=x}\right)$ replaced with $W^{*}\left(P_{\;R|X=x,Z=z}\right)$ and $W^{*}$ replaced with $W^{*}\left(P_{\;R|Z=z}\right)$ with *z* fixed, we can bound the expression in parentheses as

$$\begin{array}{c} \int W^{*}\left(P_{\;R|X=x,Z=z}\right)P_{X|Z=z}\left(dx\right)-W^{*}\left(P_{\;R|Z=z}\right)\\ \leq \int D\left(P_{\;R|X=x,Z=z}∥P_{\;R|Z=z}\right)P_{X|Z=z}\left(dx\right), \end{array}$$

and therefore

(32) $$\begin{array}{cc} W^{*}\left(X\right)-W^{*}\left(Z\right)\\ \; & \leq \int \int D\left(P_{\;R|X=x,Z=z}∥P_{\;R|Z=z}\right)P_{X|Z=z}\left(dx\right)P_{Z}\left(dz\right)\\ \; & =I(R;X|Z), \end{array}$$

where (32) follows from the alternative expression (12) of the conditional mutual information.

As in the proof Theorem 3, the conditional independence of *R* and Z=T(X) given *X* implies

I(R;X|Z)=I(R;X)−I(R;T(X)),

which completes the proof. □

### 4.4. Information Bottleneck

Let *X* and *Y* be random variables as in Section 2. When Y→X→Z, the joint distribution $P_{YXZ}$ of the triple (Y,X,Z) is determined (for fixed $P_{YX}$) by the conditional distribution (transition kernel) $P_{Z|X}$ as $P_{YXZ}=P_{YX}P_{Z|X}$. The information bottleneck (IB) framework can be formulated as the study of the constrained optimization problem

(33) $$\begin{array}{cc} \mathrm{maximize}\;\;\;\; & I(Y;Z)\\ \; & \;\\ \mathrm{subject}\;\mathrm{to}\;\;\; & I(X;Z)\leq \alpha \end{array}$$

for a given α>0, where the maximization is over all transition kernels $P_{Z|X}$.

Originally proposed by Tishby et al. [35], the solution to the IB problem is a transition kernel $P_{Z|X}$, interpreted as a stochastic transformation, that “encodes” *X* into a “compressed” representation *Z* that preserves relevant information about *Y* through maximizing I(Y;Z), while compressing *X* by requiring that I(X;Z)≤α. The intuition behind this framework is that by maximizing I(Y;Z), the representation *Z* will retain the predictive power of *X* with respect to *Y*, while the requirement I(X;Z)≤α makes the representation *Z* concise.

Note that, in case *X* is discrete and has finite entropy H(X), setting α=H(X), or setting formally α=∞ in the general case, the constraint I(X;Z)≤α becomes vacuous and (assuming the alphabet of *Z* is sufficiently large) the resulting *Z* will achieve the upper bound I(Y;Z)=I(Y;X), so that I(Y;X|Z)=I(Y;X)−I(Y;Z)=0, i.e., Y→Z→X. Thus, the solution to (33) can be considered as a stochastically relaxed version of a minimal sufficient statistic for *X* in predicting *Y* (see Goldfeld and Polyanskiy ([36], Section II.C) for more on this interpretation). Recent tutorials on the IB problem include Asoodeh and Calmon [37] and Zaidi et al. [38].

Theorem 3 and its corollaries can be used to motivate the IB principle from an estimation-theoretic viewpoint. Let

$$I\left(\alpha \right)=\underset{P_{Z|X}:I\left(X;Z\right)\leq \alpha }{\sup}I\left(Y;Z\right)$$

be the value function for (33) and $Z_{\alpha }$ a resulting optimal *Z* (assuming such a maximizer exists). From the remark after Theorem 3, we know that the bounds given in the theorem and in its corollaries remain valid if we replace T(X) with a random variable *Z*, such that Y→X→Z. Then, for example, Corollary 1 implies that

$$L_{\ell }^{*}\left(Y|Z_{\alpha }\right)-L_{\ell }^{*}\left(Y|X\right)\leq c\sqrt{I(Y;X)-I(\alpha )}$$

for all *ℓ* such that ${∥\ell ∥}_{\infty }\leq \sqrt{2}c$.

Thus, the IB paradigm minimizes, under the complexity constraint I(X;Z)≤α, an upper bound on the difference $L_{\ell }^{*}\left(Y|Z\right)-L_{\ell }^{*}\left(Y|X\right)$ that *universally holds* for all loss functions *ℓ* with ${∥\ell ∥}_{\infty }\leq \sqrt{2}c$. The resulting $Z_{\alpha }$ will then have *guaranteed performance* in predicting *Y* with respect to *all* sufficiently bounded loss functions. This gives a novel operational interpretation of the IB framework that seems to have been overlooked in the literature.

### 4.5. Deep Learning

The IB paradigm can also serve as a learning objective in deep neural networks (DNNs). Here the Lagrangian relaxation of (33) is considered. In particular, letting *X* denote the input and $Z^{\theta }$ the output of the last hidden layer of the DNN, where $\theta \in \Theta \subset R^{K}$ is the collection of network parameters (weights), the objective is to maximize

(34) $$I\left(Y;Z^{\theta }\right)-\beta I\left(X;Z^{\theta }\right)$$

over θ∈Θ for a given β>0. The parameter β controls the trade-off between how informative $Z^{\theta }$ is about *Y*, measured by $I(Y;Z^{\theta })$, and how much $Z^{\theta }$ is “compressed,” measured by $I(X;Z^{\theta })$. Clearly, larger values of β correspond to smaller values of $I(X;Z^{\theta })$ and thus to more compression. Here, $Z^{\theta }$ is either a deterministic function of *X* in the form of $Z^{\theta }=T^{\theta }\left(X\right)$, where $T^{\theta }:R^{d}\to R^{d^{\prime }}$ represents the deterministic DNN, or it is produced by a stochastic kernel $P_{Z|X}^{\theta }$, parameterized by the network parameters θ∈Θ. The latter is achieved by injecting independent noise into the network’s intermediate layers.

In addition to the motivation explained in the previous section, the IB framework for DNNs can be thought as a regularization method that results in improved generalization capabilities for a network trained on data using stochastic gradient-based methods, see, e.g., Tishby and Zaslavsky [39], Shwartz-Ziv and Tishby [40], Alemi et al. [41], as well as many other references in the excellent survey article Goldfeld and Polyanskiy [36], and the special issue [42] on information bottleneck and deep learning.

As in the previous section, our Theorem 1 and corollaries can serve as a (partial) justification for setting (34) as a learning objective. Assume that after training with a given β>0, the obtained $Z^{\theta (\beta )}$ has (true) mutual information $I(Y;Z^{\theta (\beta )})$ with *Y* (typically, this will not be the optimal solution, since maximizing (34) is not feasible and in practice only a proxy lower bound is optimized during training, see, e.g., Alemi et al. [41]). Then, by Corollary 1 the obtained network has a guaranteed predictive performance

$$L_{\ell }^{*}\left(Y|Z^{\theta (\beta )}\right)\leq L_{\ell }^{*}\left(Y|X\right)+c\epsilon$$

for all loss functions *ℓ* with ${∥\ell ∥}_{\infty }\leq \sqrt{2}c$, where

$$\epsilon =\sqrt{I\left(Y;X\right)-I\left(Y;Z^{\theta (\beta )}\right)}\;.$$

## 5. Proof of Theorem 2

**Proof Theorem** **2.**

(a) The bounds given in the proof of Theorem 1 in [2] imply
  $$\begin{array}{cc} L_{n}\; & \leq J_{n,1}+J_{n,2}+J_{n,3}+J_{n,4}+J_{n,5}, \end{array}$$
  where
  $$\begin{array}{ll} J_{n,1}\; & =\sum_{A\in P_{n},B\in Q_{n},C\in R_{n}}\left|P_{XYZ}^{n}\left(A,B,C\right)-P_{XYZ}\left(A,B,C\right)\right|,\\ J_{n,2}\; & =\sum_{B\in Q_{n},C\in R_{n}}\left|P_{YZ}\left(B,C\right)-P_{YZ}^{n}\left(B,C\right)\right|,\\ J_{n,3} & =\sum_{A\in P_{n},C\in R_{n}}\left|P_{XZ}\left(A,C\right)-P_{XZ}^{n}\left(A,C\right)\right|,\\ J_{n,4}\; & =\sum_{C\in R_{n}}\left|P_{Z}^{n}\left(C\right)-P_{Z}\left(C\right)\right|, \end{array}$$
  and
  $$\begin{array}{cc} J_{n,5}\; & =\sum_{A\in P_{n},B\in Q_{n},C\in R_{n}}\left|P_{XYZ}\left(A,B,C\right)-\frac{P_{XZ}\left(A,C\right)P_{YZ}\left(B,C\right)}{P_{Z}\left(C\right)}\right|\;. \end{array}$$
  Using large deviation inequalities from Beirlant et al. [43] and in Biau and Györfi [18], Györfi and Walk [2] proved that for all $\varepsilon_{i}>0$, i=1,⋯,4 and δ>0,
  (35) $$\begin{array}{ll} P(L_{n}>\varepsilon_{1}+\varepsilon_{2}+\varepsilon_{3}+\varepsilon_{4}+\delta )\\ \; & \leq P\left(J_{n,1}>\varepsilon_{1}\right)+P\left(J_{n,2}>\varepsilon_{2}\right)+P\left(J_{n,3}>\varepsilon_{3}\right)+P\left(J_{n,4}>\varepsilon_{4}\right)+I_{J_{n,5}>\delta }\\ \; & \leq 2^{m_{n}\cdot m_{n}^{\prime }\cdot m_{n}^{\prime \prime }}e^{-n\varepsilon_{1}^{2}/2}+2^{m_{n}^{\prime }\cdot m_{n}^{\prime \prime }}e^{-n\varepsilon_{2}^{2}/2}+2^{m_{n}\cdot m_{n}^{\prime \prime }}e^{-n\varepsilon_{3}^{2}/2}+2^{m_{n}^{\prime \prime }}e^{-n\varepsilon_{4}^{2}/2}\\ \; & \;+I_{J_{n,5}>\delta }. \end{array}$$
  We note that the bounds on the probabilities $P(J_{n,i}>\varepsilon_{i})$ for i=1,…,4 were proven in [2] without either assuming the null hypothesis $H_{0}$ or using the condition that the partitions are nested. Under the null hypothesis, Györfi and Walk [2] claimed that
  $$\begin{array}{c} J_{n,5}=0. \end{array}$$
  As Neykov et al. [12] observed, this was incorrect. In order to resolve the gap, we show that under $\lim_{n}h_{n}=0$ and condition (5) and under the null hypothesis, the last term in (35) is o(1), i.e.,
  $$\begin{array}{c} I_{\sum_{A\in P_{n},B\in Q_{n},C\in R_{n}}\left|P_{XYZ}\left(A,B,C\right)-\frac{P_{XZ}\left(A,C\right)P_{YZ}\left(B,C\right)}{P_{Z}\left(C\right)}\right|>\delta }=0 \end{array}$$
  if *n* is large enough. The null hypothesis implies that
  $$\begin{array}{ll} P_{XYZ}\left(A,B,C\right)-\frac{P_{XZ}\left(A,C\right)P_{YZ}\left(B,C\right)}{P_{Z}\left(C\right)}\\ \; & =P\left(X\in A,Y\in B,Z\in C\right)-\frac{P_{XZ}\left(A,C\right)P\left(Y\in B,Z\in C\right)}{P_{Z}\left(C\right)}\\ \; & =E\left[P\left(X\in A,Y\in B|Z\right)I_{Z\in C}\right]-E\left[P\left(Y\in B|Z\right)I_{Z\in C}\right]\frac{P_{XZ}\left(A,C\right)}{P_{Z}\left(C\right)}\\ \; & =E\left[P\left(X\in A|Z\right)P\left(Y\in B|Z\right)I_{Z\in C}\right]-E\left[P\left(Y\in B|Z\right)I_{Z\in C}\right]\frac{P_{XZ}\left(A,C\right)}{P_{Z}\left(C\right)}. \end{array}$$
  Thus,
  $$\begin{array}{cc} \sum_{A\in P_{n},B\in Q_{n},C\in R_{n}}\left|P_{XYZ}\left(A,B,C\right)-\frac{P_{XZ}\left(A,C\right)P_{YZ}\left(B,C\right)}{P_{Z}\left(C\right)}\right|\\ \; & \leq \sum_{A\in P_{n},B\in Q_{n},C\in R_{n}}E\left[P\left(Y\in B|Z\right)\left|P\left(X\in A|Z\right)I_{Z\in C}-I_{Z\in C}\frac{P_{XZ}\left(A,C\right)}{P_{Z}\left(C\right)}\right|\right]\\ \; & =\sum_{A\in P_{n},C\in R_{n}}E\left[\left|P\left(X\in A|Z\right)I_{Z\in C}-I_{Z\in C}\frac{P_{XZ}\left(A,C\right)}{P_{Z}\left(C\right)}\right|\right]. \end{array}$$
  Let p(·∣z) and $C_{n}\left(z\right)$ be as in Condition 1. Then,
  (36) $$\begin{array}{ll} \sum_{A\in P_{n},C\in R_{n}}E\left[\left|P\left(X\in A|Z\right)I_{Z\in C}-I_{Z\in C}\frac{P_{XZ}\left(A,C\right)}{P_{Z}\left(C\right)}\right|\right]\\ \; & =\sum_{A\in P_{n},C\in R_{n}}\int_{C}\left|\int_{A}p\left(x|z\right)P_{X}\left(dx\right)-\frac{\int_{A}\left[\int_{C}p\left(x|z^{\prime }\right)P_{Z}\left(dz^{\prime }\right)\right]P_{X}\left(dx\right)}{P_{Z}\left(C\right)}\right|P_{Z}\left(dz\right)\\ \; & \leq \int \int \left|p\left(x|z\right)-\frac{\int_{C_{n}\left(z\right)}p\left(x|z^{\prime }\right)P_{Z}\left(dz^{\prime }\right)}{P_{Z}\left(C_{n}\left(z\right)\right)}\right|P_{Z}\left(dz\right)P_{X}\left(dx\right)\\ \; & \leq C^{*}h_{n}, \end{array}$$
  where in the last step we use condition (5). The inequalities (35) and (36) imply that
  $$\begin{array}{ll} P\left(L_{n}>c_{1}\left(\sqrt{\frac{m_{n}m_{n}^{\prime }m_{n}^{\prime \prime }}{n}}+\sqrt{\frac{m_{n}^{\prime }m_{n}^{\prime \prime }}{n}}+\sqrt{\frac{m_{n}m_{n}^{\prime \prime }}{n}}+\sqrt{\frac{m_{n}^{\prime \prime }}{n}}\;\right)+\left(\log n\right)h_{n}\right)\\ \; & \leq 4e^{-\left(c_{1}^{2}/2-\log2\right)m_{n}^{\prime \prime }}+I_{C^{*}h_{n}>\left(\log n\right)h_{n}}\\ \; & \leq 4e^{-\left(c_{1}^{2}/2-\log2\right)m_{n}^{\prime \prime }}, \end{array}$$
  if $n\geq e^{C^{*}}$. Since $m_{n}^{\prime \prime }$ is proportional to $1/h_{n}^{d^{\prime }}$, condition (8) on $h_{n}$ implies $\sum_{n=1}^{\infty }P\left(L_{n}\geq t_{n}\right)<\infty$, and thus using the Borel–Cantelli lemma, after a random sample size, the test has no error with a probability of one.
(b) This proof is a refinement of the proof of Corollary 1 in [2], in which we avoid the condition used there that the sequences of partitions $P_{n}$ and $Q_{n}$ are nested. According to the proof of Part (a) (see the remark after (35)), we obtain that
  $$\underset{n\to \infty }{lim inf}L_{n}\geq \underset{n\to \infty }{lim inf}\left(L_{n}-J_{n,5}\right)+\underset{n\to \infty }{lim inf}J_{n,5}=\underset{n\to \infty }{lim inf}J_{n,5}\;a.s.$$
  To simplify the notation, let $P_{XY|z}=P_{XY|Z=z}$, $P_{X|z}=P_{X|Z=z}$, and $P_{Y|z}=P_{Y|Z=z}$. Let $L^{*}$ be the expected total variation distance between $P_{XY|z}$ and $P_{X|z}P_{Y|z}\;$:
  $$L^{*}=\int \underset{F}{\sup}|P_{XY|z}\left(F\right)-P_{X|z}P_{Y|z}\left(F\right)|\;P_{Z}\left(dz\right),$$
  where the supremum is taken over all Borel subsets *F* of $R^{d}\times R^{d^{\prime }}$. It suffices to prove that using the condition $\lim_{n}h_{n}=0$,
  $$\underset{n\to \infty }{lim inf}J_{n,5}\geq 2L^{*}>0.$$
  One has that
  $$\begin{array}{r} \sum_{A\in P_{n},B\in Q_{n},C\in R_{n}}\left|P_{XYZ}\left(A,B,C\right)-\frac{P_{XZ}\left(A,C\right)P_{YZ}\left(B,C\right)}{P_{Z}\left(C\right)}\right|\\ \;\;\;\;\;\;\;\;\geq \int \sum_{A\in P_{n},B\in Q_{n}}\left|P_{XY|z}\left(A,B\right)-P_{X|z}\left(A\right)P_{Y|z}\left(B\right)\right|P_{Z}\left(dz\right)-W_{n}, \end{array}$$
  where
  (37) $$\begin{array}{cc} \;\;\;\;W_{n}\; & \leq \sum_{A\in P_{n},B\in Q_{n}}\int \left|\frac{\int_{C_{n}\left(z\right)}P_{XY|z^{\prime }}\left(A,B\right)P_{Z}\left(z^{\prime }\right)}{P_{Z}\left(C_{n}\left(z\right)\right)}-P_{XY|z}\left(A,B\right)\right|P_{Z}\left(dz\right) \end{array}$$
  (38) $$\begin{array}{cc} \; & \;+\sum_{A\in P_{n}}\int \left|\frac{\int_{C_{n}\left(z\right)}P_{X|z^{\prime }}\left(A\right)P_{Z}\left(dz^{\prime }\right)}{P_{Z}\left(C_{n}\left(z\right)\right)}-P_{X|z}\left(A\right)\right|P_{Z}\left(dz\right) \end{array}$$
  (39) $$\begin{array}{cc} \; & \;+\sum_{B\in Q_{n}}\int \left|\frac{\int_{C_{n}\left(z\right)}P_{Y|z^{\prime }}\left(B\right)P_{Z}\left(dz^{\prime }\right)}{P_{Z}\left(C_{n}\left(z\right)\right)}-P_{Y|z}\left(B\right)\right|P_{Z}\left(dz\right) \end{array}$$
  In [2], it was shown that the condition $\lim_{n}h_{n}=0$ implies $\lim_{n}W_{n}=0$ if the sequence of partitions ${\left\{P_{n},Q_{n}\right\}}_{n\geq 1}$ is nested. In order to avoid this nestedness condition, introduce the density p(x,y|z) of the conditional distribution $P_{XY|z}$ with respect to the distribution $P_{XY}$ of (X,Y) as a dominating measure, and similarly let $p_{n}\left(x,y|z\right)$ be the density of the conditional distribution $\int_{C_{n}\left(z\right)}P_{XY|z^{\prime }}\left(\;\cdot \;,\;\cdot \;\right)P_{Z}\left(dz^{\prime }\right)/P_{Z}\left(C_{n}\left(z\right)\right)$ with respect to $P_{XY}$, i.e., $p_{n}\left(x,y|z\right)=\int_{C_{n}\left(z\right)}p\left(x,y|z^{\prime }\right)P_{Z}\left(dz^{\prime }\right)/P_{Z}\left(C_{n}\left(z\right)\right)$. Then,
  $$\begin{array}{ll} \sum_{A\in P_{n},B\in Q_{n}}\left|\frac{\int_{C_{n}\left(z\right)}P_{XY|z^{\prime }}\left(A,B\right)P_{Z}\left(dz^{\prime }\right)}{P_{Z}\left(C_{n}\left(z\right)\right)}-P_{XY|z}\left(A,B\right)\right|\\ \; & \leq \int \int |p_{n}\left(x,y|z\right)-p\left(x,y|z\right)|P_{XY}\left(dx,dy\right), \end{array}$$
  and therefore the term on the right-hand side of (37) will converge to zero, as long as
  $$\begin{array}{c} \int \int \int |p_{n}\left(x,y|z\right)-p\left(x,y|z\right)|P_{XY}\left(dx,dy\right)P_{Z}\left(dz\right)\to 0, \end{array}$$
  which follows from $\lim_{n}h_{n}=0$ using the standard technique of the bias of partitioning regression estimate for the regression function p(·,·|z); see Theorem 4.2 in [44]. The terms in (38) and (39) can be dealt with analogously. Thus,
  $$\begin{array}{cc} \underset{n\to \infty }{lim inf}J_{n,5}\; & \geq \underset{n\to \infty }{lim inf}\int \sum_{A\in P_{n},B\in Q_{n}}\left|P_{XY|z}\left(A,B\right)-P_{X|z}\left(A\right)P_{Y|z}\left(B\right)\right|P_{Z}\left(dz\right). \end{array}$$
  For fixed *z*, $\lim_{n}h_{n}=0$ implies
  $$\begin{array}{l} \lim_{n\to \infty }\sum_{A\in P_{n},B\in Q_{n}}\left|P_{XY|z}\left(A,B\right)-P_{X|z}\left(A\right)P_{Y|z}\left(B\right)\right|\\ \;\;\;\;\;\;=2\underset{F}{\sup}|P_{XY|z}\left(F\right)-P_{X|z}P_{Y|z}\left(F\right)|, \end{array}$$
  see Abou-Jaoude [45] and Csiszár [46]. Therefore, the dominated convergence theorem yields
  $$\begin{array}{l} \lim_{n\to \infty }\int \;\;\;\sum_{A\in P_{n},B\in Q_{n}}\left|P_{XY|z}\left(A,B\right)-P_{X|z}\left(A\right)P_{Y|z}\left(B\right)\right|P_{Z}\left(dz\right)\\ \;\;\;\;\;\;=2\int \underset{F}{\sup}|P_{XY|z}\left(F\right)-P_{X|z}P_{Y|z}\left(F\right)|\;P_{Z}\left(dz\right)\\ \;\;\;\;\;\;=2L^{*}. \end{array}$$

□

Note that in the proof of Part (b) the condition (5) is not used, at all.

## 6. Concluding Remarks

We studied the excess minimum risk in statistical inference and under mild conditions gave a strongly consistent procedure for testing from data if a given transformation of an observed feature vector results in zero excess minimum risk for all loss functions. It is an open research problem whether a strong universal test exists, i.e., a test that is strongly consistent without any conditions on the transformation and on the underlying distribution. We also developed information-theoretic upper bounds on the excess risk that uniformly hold over fairly general classes of loss functions. The bounds were not stated in the most general form possible, in that the observed quantities were restricted to taking values in Euclidean spaces and we did not allow transformations that were random functions of the observation, both of which restrictions could be relaxed.The bounds could be sharpened, e.g., in specific cases, but in their present form are already useful. For example, they give an additional theoretical motivation for applying the information bottleneck approach in deep learning.

## Data Availability

Not applicable.
